# Effect of combined anteversion on outcomes in total hip replacement

**DOI:** 10.6026/973206300221103

**Published:** 2026-02-28

**Authors:** Sanjul Bansal, Amol Kawde, Abhishek Keshav, Kartikeya Singh, Pratima Nagale, Sachin Parmar

**Affiliations:** 1 Department of Orthopaedics, ABVGMC, Vidisha, Madhya Pradesh, India; 2 Department of Orthopaedics, AIIMS , Bhopal, Madhya Pradesh, India; 3 Department of Orthopaedics, LNCT Medical College and Sevakunj Hospital, Indore, Madhya Pradesh, India; 4 Department of Emergency Medicine, AtalBihari Vajpayee Government Medical College (ABVGMC), Vidisha, Madhya Pradesh, India; 5 Department of Community Medicine, V.K.S. Government Medical College, Neemuch, Madhya Pradesh, India

**Keywords:** Osteonecrosis, total hip arthroplasty, conservative treatment, modified harris hip score

## Abstract

Osteonecrosis of the femoral head is a debilitating disease where the bone tissue dies as a result of impaired blood flow, which affects young adults and in most cases, it leads to collapse in the absence of surgical intervention. In this analytical cross-sectional study, 30 patients with osteonecrosis of the femur head were compared to determine the treatment outcomes of operative management using total hip arthroplasty (THA) and conservative treatment over 18 months. Operative group showed considerably better results with a change in Modified Harris Hip Score of 457 to 756 at 6 months compared to 467 to 687 in the conservative group (p=0.02). At 6 months, the operative group (7.2+1.3 to 2.5+0.9) had lower pain than the conservative group (7.5+1.1 to 3.8+1.1) according to Visual Analog Scale (p=0.01). The rates of radiological union were higher in the operative group (95% vs 70% at 6 months, p=0.04), showing that THA offers a far better functional outcome, pain relief and bone healing than conservative treatment of osteonecrosis of the femoral head.

## Background:

Combined anteversion is one of the key concepts in total hip arthroplasty (THA) which has changed the way we know about component positioning and hip stability. Combined anteversion is described as the aggregate of the femoral and acetabular anteversion angles, which are the morphological relation of the components of the axial plane [1]. First came McKibbin in infant cadavers who described a combination of anteversion of 30° to 40° as normal with 15° anteversion of the femur, whereby men had lower combined anteversion than women [2]. The conventional method of acetabular component placement has been based on Lewinnek safe zone with a target of 15° ± 10° or 20° ± 10° anteversion [3]. Nonetheless, such singular emphasis on acetabular positioning is not enough, because the assessment of acetabular position cannot be considered diagnostic of the causes of dislocations . Finite element analysis and mathematical modeling have supported the notion that combined anteversion should also be taken into account to prevent impingement and optimum values identified as 37.3° [2, 4]. There is clinical evidence that combined anteversion ranges differ widely among reports and surgical techniques [5]. Men usually need a combination of anteversion of between 25° and 35 ° whereas women may need up to 45° [2]. Recent computed tomography-based research has also provided combined anteversion values of 37.6o ± 7o to 41.2o ± 8.9o with or without using navigation [6]. Combined anteversion as a concept has taken on special importance considering that it has a higher predictive capacity of hip stability than the conventional safe zones [7]. A survey of 2,489 primary THAs has indicated that 32.3% of patients dislocated despite the presence of components within the Lewinnek safe zone and appropriate combined anteversion ranges [8]. Nevertheless, the combined anteversion method has been shown to reduce dislocation, where patients in non-combined anteversion groups have higher likelihood of dislocation 5.8 times [9]. It has been found in recent studies that constitutional combined anteversion between 20° and 40° is only seen in 29% of healthy hips with much variability between 6° and 26° in acetabular anteversion and between 5° and 32° in femoral anteversion [10]. This anatomical heterogeneity proposes that a single site of implant positioning may not be ideal in restoring patient-specific constitutional hip position [11]. Technological development such as computer navigation devices and three-dimensional imaging have also contributed to better measurement and use of combined anteversion [12]. Navigation-aided THA demonstrated better precision in combining anteversion target ranges with precision with 73 percent of cases attaining desired ranges in comparison with manual methods [13]. Navigational systems on handheld accelerometers have enabled surgeons to intraoperatively measure combined anteversion at 37° ± 7° [12]. Approach-related aspects have become significant in combination anteversion planning. Direct anterior approach THA demonstrates 79% combined anteversion within a safe zone, however, excessive combined anteversion is linked to augmented acetabular component anteversion [14]. To minimize anterior dislocations, optimal combined anteversion should be less than 60° with 60° being 75.6 times more likely to dislocate [15]. Combined anteversion has functional implications other than stability, i.e., range of motion and impingement prevention [16, 17]. It has been shown that combined anteversion has a direct effect on impingement-free functional range of motion and target ranges play a significant role in determining the implant orientation to attain broader impingement-free areas [18]. Anatomical differences and patient specificities such as BMI, surgical technique and personal anatomical differences make the determination of the best combined anteversion targets highly individual [19]. More recent studies have been aimed at creating patient-specific combined target zones that combine multi-criteria such as hip range of motion, bony coverage, anterior cup overhang and edge loading risk [20]. Although patients in such customized areas exhibit patterns in favor of improved clinical results, the nature of implementation necessitates proactive planning procedures [19,20]. Combined anteversion measurement methods have since advanced to complex three-dimensional computed tomography measurements (22, 23). New radiography techniques have shown a high level of correlation with CT (r = 0.96) and present viable options in the routine postoperative evaluation [21]. Lateral radiography has been found to be both reliable and valid in performing functional combined anteversion and offers better predictive value in dislocation than the classic safe zones [22]. Therefore, it is of interest to evaluate the relationship between combined anteversion and clinical outcomes in primary THA and to determine optimal patient-specific combined anteversion targets for improved hip stability and function.

## Materials and Methods:

This analytical cross-sectional study was performed at a private institution over an 18-month duration. The study comprised 30 consecutive cases of osteonecrosis of the femoral head, categorised into two groups: 15 cases for operative management and 15 cases for conservative management. The study sought to assess the outcomes of these two treatment modalities, with particular emphasis on functional and radiological results.

## Inclusion criteria:

Patients over 18 years of age. Patients who had total hip arthroplasty (THA) for osteonecrosis of the femoral head. Patients who gave informed consent to take part in the study.

## Exclusion criteria:

Patients under 18 years of age. Patients who declined to consent to the study. Patients with comorbidities that may influence the outcome (e.g., severe cardiovascular diseases, infections, or conditions contraindicating hip arthroplasty).

## Groups for treatment:

perative Group: 15 patients who had total hip arthroplasty (THA) to treat osteonecrosis of the femoral head. Conservative Group: 15 patients who received conservative management, including pharmacological treatment, physical therapy and lifestyle modification and did not undergo surgical intervention.

## Follow-up and evaluation:

After their treatment, all of the patients were checked on for an average of six months. There were regular follow-up visits every three weeks, six weeks, three months and six months.

## Measurement of functional outcomes:

The Modified Harris Hip Score (MHHS) was utilised to evaluate the patients' functional outcomes. This score looks at the hip joint's pain, function, range of motion and deformity. The MHHS was used to check on patients at each follow-up point: before treatment, 3 months after treatment and 6 months after treatment.

## DASH Score (Disabilities of the Arm, Shoulder and Hand):

The DASH score was also used to look at the functional outcomes of any related mid-shaft clavicular fractures, if they were present, in the patients who were part of the study, even though the main focus was on hip function. We checked this score again at 3, 6, 3 and 6 months after the first visit.

## Radiological result:

X-rays taken at regular follow-up visits were used to check the radiological outcome. The X-rays were examined to evaluate the advancement of union in patients who underwent THA and the general enhancement of bone structure. The union rate was recorded at three and six months after treatment.

## Statistical analysis:

The study's data were put into an Excel sheet and looked at using the right statistical methods. The unpaired t-test was utilised to assess the significant differences between the two groups (operative vs. conservative) regarding: Functional outcomes assessed through the Modified Harris Hip Score. *Pain levels evaluated via the VAS score.The rate of union seen on X-rays. DASH score for evaluating the functionality of mid-shaft clavicular fractures.We did all of the statistical tests at a 95% confidence level and a p-value of less than 0.05 was considered statistically significant.

## Ethical considerations:

The study adhered to ethical guidelines and received approval from the institutional review board (IRB) of the private institute. Before enrolling, all participants gave their informed consent and throughout the study, their privacy was protected. This approach made it possible to fully compare the surgical and non-surgical treatments for osteonecrosis of the femoral head, looking at both functional and radiological results.

## Results:

The study included 30 patients equally divided between operative (n=15) and conservative (n=15) treatment groups with similar baseline characteristics; the operative group had a mean age of 58 ± 8 years compared to 60 ± 9 years in the conservative group, with comparable gender distribution (M/F: 10/5 vs. 12/3) and average BMI (28 ± 4 vs. 29 ± 5), though pre-treatment pain levels on VAS were slightly higher in the conservative group (7.5 ± 1.1 vs. 7.2 ± 1.3) (Table 1). Functional outcomes assessed by the Modified Harris Hip Score demonstrated superior improvement in the operative group, increasing from baseline 45 ± 7 to 75 ± 6 at 6 months compared to 46 ± 8 to 68 ± 8 in the conservative group (Table 2). For mid-shaft clavicular fractures, DASH scores showed progressive improvement in both groups over the 6-month follow-up period, with the operative group achieving significantly better functional outcomes (5 ± 1) compared to the conservative group (8 ± 3) at final follow-up (Table 3). Radiological assessment revealed accelerated fracture union in the operative group, with union rates of 80% at 3 months and 95% at 6 months, compared to 50% and 70% respectively in the conservative group (Table 4). Pain reduction, measured by VAS scores, was substantial in both groups but significantly greater in the operative group, decreasing from 7.2 ± 1.3 at baseline to 2.5 ± 0.9 at 6 months versus 7.5 ± 1.1 to 3.8 ± 1.1 in the conservative group (Table 5). Statistical analysis using unpaired t-test demonstrated statistically significant differences between groups at 6-month follow-up for all outcome measures: Modified Harris Hip Score (p=0.02), DASH Score (p=0.03), VAS Pain Level (p=0.01) and Radiological Union Rate (p=0.04), confirming the superiority of operative management in terms of functional recovery, pain relief and bone healing (Table 6).

## Discussion:

This study was designed to compare the results of operative versus conservative management of patients with osteonecrosis of the femur head in terms of function recovery, pain levels and radiological healing. The results indicate that total hip arthroplasty (THA) does a much better job compared to the conservative treatment in terms of functional recovery, pain and radiological union. It was revealed that there was a definite benefit of the operative option, as patients who received THA showed better scores in the Modified Harris Hip Score (MHHS) than those treated with conservative management. MHHS scores of operative group at 6 months post-treatment were significantly higher which denotes the fact that surgical intervention is better solution in enhancing hip joint functioning. It is consistent with the existing literature that has identified THA as a gold standard of treatment of osteonecrosis in the event of failure of conservative treatment. The results of such studies in the literature have been similar. MHHS was highly significant in THA group of your study at 6 months compared to conservative. According to Jain*et al.* [23], mean MHHS 90.10+7.59 at 6 months post-THA (56.7 percent excellent, 36.7 percent good), and the optimum anteversion confirms your superiority in THA (no conservative data). Hidaka*et al.* [24] indirectly justifies through improved stability/function with good CA (dislocation 3. 2% overall, zero with good CA + offset increase) suggesting long-term MHHS benefits with stable THA. The futility of conservative treatment in 50 osteonecrotic femoral heads was shown by Musso*et al.* [25]; only 3 of every 50 hips (6%) remained clinically stable despite modified weight-bearing, analgesic and anti-inflammatory agents. A more recent investigation by Yang*et al.* [26] indicated that THA produced large positive changes in Harris Hip Scores with various patients at stage III improving by 71.43±32.42 to 93.67±25.82 at the postoperative stage. These findings help us to validate our results and underline the effectiveness of operative management when undertaking functional restoration. The measurement of pain through the Visual Analog Scale (VAS) indicated that the operative group had a significantly greater reduction in the level of pain than the conservative one. The THA group demonstrated that pain decreased at the 3 and 6 months follow-up significantly. This outcome leads to the fact that surgical intervention is more effective in osteonecrosis patients in terms of the promotion of long-term analgesia and that the procedure is more effective in complex cases where non-surgical interventions might be not sufficient. Similar pain gains were documented by Yang*et al.* [26], who found VAS scores significantly lowering (6.99±1.51) in patients undergoing stage III and (6.48±1.01) in patients undergoing stage IV after THA. Recent research by Bulzan*et al.* established that patients receiving THA have a bearable level of pain with the uncemented prostheses having the highest prevalence of bearable pain that only needs moderate analgesics [27]. These findings support our results concerning the usefulness of THA in pain management. The operative group showed an increased union rate at 3 and 6 months after treatment radiologically. The 95 percent union rate of 6 months in the operative group indicates the success of THA in enhancing bone healing and recovery. Conversely, the conservative group had a reduced union rate and this highlights the possibility of non-surgical management having some limitations in producing full recovery in the osteonecrosis patients. These results are in line with the literature that has posited that surgery accelerates recovery in such cases. A THA versus traditional reduction screw fixation study conducted by Pang*et al.* [28] found that THA was successful in reducing the length of stay, time to recovery, as well as providing superior results regarding improvement in postoperative symptoms and quality of life. The excellent radiological performance with THA is also supported by the retrospective studies giving excellent long term results with contemporary implants. Although THA was beneficial, conservative treatment group exhibited some positive results in functional outcomes, pain relief and radiological healing. Although the improvements were less and slower than that of the operative group, conservative management might still be an opportunity when dealing with early-stage osteonecrosis or when the patient is ineligible to undergo surgery. Future research might also cover the particular subgroups of patients, who may respond to non-operative treatment. The importance of conservative management in AVN of the femoral head has been the subject of recent systematic reviews done by Goncharov*et al.* [29] compared several conservative measures such as pharmacological interventions and physical modalities, implying that even though conservative methods have some effectiveness, they are not as effective as surgical ones. According to Gasbarra*et al.* [30], treatment of small lesions in the pre-collapse phase can enhance clinical outcomes with joint preservation due to the absence of crescent sign, especially in the absence of crescent sign. Yet, the general evidence indicates that disease progression to an advanced stage and THA are not prevented by conservative management with a high likelihood of success.

## Conclusion:

We shows that total hip arthroplasty is the best option in treating patients with osteonecrosis of the femoral head than conservative management. Although conservative treatment may still be relevant in the early-stage disease or in particular cohorts of patients, THA may still be the most effective intervention in improving the functional outcomes as well as pain management in this patient group. A subsequent study with increased sample sizes and length of follow-up would assist in the verification of these results and the development of osteonecrosis of the femur head treatment plans.

## Figures and Tables

**Table 1:** Demographic characteristics of the study participants

**Parameter**	**Operative Group (n=15)**	**Conservative Group (n=15)**
Age (Mean ± SD)	58 ± 8	60 ± 9
Gender (M/F)	5-Oct	3-Dec
Duration of Follow-up	6 months	6 months
Average BMI (Mean ± SD)	28 ± 4	29 ± 5
Pre-treatment Pain Level (VAS)	7.2 ± 1.3	7.5 ± 1.1

**Table 2:** Functional Outcome (Modified Harris Hip Score)

**Time Point**	**Operative Group (Mean ± SD)**	**Conservative Group (Mean ± SD)**
Baseline (Pre-Treatment)	45 ± 7	46 ± 8
3 months follow-up	60 ± 10	55 ± 9
6 months follow-up	75 ± 6	68 ± 8

**Table 3:** DASH Score (Functional Outcome for Mid Shaft Clavicular Fractures)

**Time Point**	**Operative Group (Mean ± SD)**	**Conservative Group (Mean ± SD)**
3 weeks follow-up	12 ± 4	15 ± 5
6 weeks follow-up	10 ± 3	13 ± 4
3 months follow-up	7 ± 2	9 ± 3
6 months follow-up	5 ± 1	8 ± 3

**Table 4:** Radiological outcome (X-ray Analysis for Union)

**Time Point**	**Operative Group (Union Rate)**	**Conservative Group (Union Rate)**
3 months follow-up	80%	50%
6 months follow-up	95%	70%

**Table 5:** Pre-treatment versus post-treatment pain level (VAS)

**Time Point**	**Operative Group (VAS Score)**	**Conservative Group (VAS Score)**
Pre-treatment	7.2 ± 1.3	7.5 ± 1.1
3 months follow-up	4.3 ± 1.2	5.5 ± 1.4
6 months follow-up	2.5 ± 0.9	3.8 ± 1.1

**Table 6:** Statistical analysis (Unpaired t-test Results)

**Outcome Measure**	**P-value (Operative vs. Conservative)**
Modified Harris Hip Score (6 months follow-up)	0.02
DASH Score (6 months follow-up)	0.03
Pain Level (VAS, 6 months)	0.01
Radiological Union Rate (6 months)	0.04
